# Inhibitory role of S-nitrosoglutathione in the aggregation of frozen platelets, and its effect on the expression of membrane glycoproteins

**DOI:** 10.3892/etm.2013.1221

**Published:** 2013-07-12

**Authors:** TAO WU, CHANGHONG ZHANG, ZANTAO WANG, JINGHAN LIU, HUI LI, WU ZHOU, SHUYING WANG

**Affiliations:** 1 Department of Blood Transfusion, General Hospital of Beijing Military Region, Beijing 100700, P.R. China; 2 Department of Surgery, Liaohe Oilfield General Hospital, Panjin, Liaoyang 124010, P.R. China; 3 Department of Blood Transfusion, Chinese PLA General Hospital, Haidian, Beijing 100853, P.R. China

**Keywords:** S-nitrosoglutathione, frozen platelets, aggregation, nitric oxide, membrane glycoprotein molecules

## Abstract

The aim of this study was to explore the effects of S-nitrosoglutathione (GSNO) on the aggregation of frozen platelets, the platelet nitric oxide (NO) content and the expression of membrane glycoproteins. The level of frozen platelet aggregation was measured using a platelet aggregation analyzer, while the content of NO was measured by the nitrate reductase method and the expression of frozen platelet membrane glycoproteins was determined by flow cytometry. The level of frozen platelet aggregation was reduced from 35.47±2.93 to 24.43±3.07% following treatment with GSNO. The mean NO concentration in the 32 samples of frozen platelets treated with GSNO was 45.64±6.31 μmol/l, which was significantly higher compared with the concentration in the fresh liquid platelet group. There were no significant differences in the levels of PAC-1 in the fresh liquid platelet, frozen platelet and GSNO-treated frozen platelet groups; by contrast, significant differences were observed in the CD42b and CD62P levels. The platelet membrane glycoprotein expression levels in the frozen platelet and the GSNO-treated frozen platelet groups were not significantly different. The results of the study indicate that GSNO has potential as a cryoprotectant, due to its ability to increase the NO concentration in frozen platelets, inhibit platelet aggregation and maintain platelet function. It is likely that the molecular arrangement and structure of the frozen platelets were altered following GSNO treatment, or that the frozen platelets were affected by alternative mechanisms.

## Introduction

Platelet cryopreservation technology has been an area of particular interest for many years. Cryoprotectants and various additives are necessary for platelets undergoing cryopreservation, in order to prevent the platelet membrane from undergoing damage during cryopreservation and affecting the normal function of the platelets following rewarming. Cryoprotectants may function by increasing the concentration of solute in the cells and reducing the number of ice crystals formed at any temperature. Common cryoprotectants included glycerol, dimethylsulfoxide (DMSO), ethylene glycol and propylene glycol.

DMSO has been demonstrated to be superior to other cryoprotectants with regard to the protective effect it exerts on frozen platelets (1). The addition of S-nitrosoglutathione (GSNO) to frozen platelets is able to inhibit platelet activation and maintain their aggregation activity, indicating the potential of GSNO as a platelet cryoprotectant (2). The addition of GSNO (a type of stable nitrosothiol) as a nitric oxide (NO) donor to frozen platelets as a supplement, when DMSO alone is insufficient, may prevent platelet consumption and maintain the function of the platelets.

NO levels are an important factor in blood, particularly with regard to the efficacy and safety of red blood cells stored for use in transfusion. The loss of NO in stored blood has become a concern in blood transfusion safety. NO may inhibit platelet function, primarily by raising the levels of cyclic guanosine monophosphate (cGMP). However, non-cGMP-dependent mechanisms, such as S-nitrosylation, have also been suggested as alternative NO-mediated signaling pathways (3). The application of NO in platelet preservation was investigated in a study by Wong and Li, in which an NO precursor was added to platelets for the *in vivo* synthesis of NO using nitric oxide synthetase (NOS); in addition, a certain quantity of NO solution directly injected into the platelets was observed to improve the platelet function (4).

Platelet membrane glycoproteins are specific glycoprotein components that are located inside the platelet membrane, on the membrane surface and in the plasma. Platelet membrane glycoproteins are important in initial hemostasis, platelet adhesion to the extracellular matrix and the subsequent platelet aggregation process. A lack of platelet membrane glycoproteins may lead to platelet dysfunction. Platelet function may be accurately detected using flow cytometry and various monoclonal antibodies specific to platelet membrane glycoproteins (5).

Several *in vitro* functional experiments have revealed defects in frozen platelets, although the *in vivo* hemostatic function of the frozen platelets has been observed to be markedly enhanced. Furthermore, the immediate hemostatic function of frozen platelets has been demonstrated to be significantly improved in comparison with that of liquid-stored platelets (1). Further studies are required to confirm whether the significant enhancement of the *in vivo* hemostatic function of the cryopreserved platelets is correlated with changes in the platelet membrane glycoproteins, and whether the GSNO-mediated inhibition of cryopreserved platelet aggregation is associated with changes in NO levels and with alterations in the platelet membrane glycoproteins.

In the present study, we detected the levels of NO in fresh liquid platelets by the nitrate reductase method. In addition, the aggregation rate and NO content of frozen platelets were monitored and compared, prior to and following the addition of GSNO. Furthermore, in order to explore the possible mechanism leading to the significantly enhanced *in vivo* hemostatic function of cryopreserved platelets, and the inhibitory effect of GSNO on platelet aggregation, we studied the expression of platelet membrane glycoproteins prior to and following platelet cryopreservation, and following treatment with GSNO.

## Materials and methods

### Specimen collection

Platelets were collected from donors by platelet apheresis, in accordance with the medical standards of blood donation in China. The donors did not take aspirin or any similar anti-platelet/anticoagulant drugs within 2 weeks prior to the donation. The peripheral blood platelet count of the samples was >1.5×10^11^/l. A total of 32 platelet apheresis donations complying with the previously mentioned conditions were randomly selected, 12 cases of which were used to determine the platelet count, membrane glycoprotein expression and aggregation rates. The study was conducted in accordance with the Declaration of Helsinki, and with approval from the Ethics Committee of the Chinese PLA General Hospital (Beijing, China). Written informed consent was obtained from all participants.

### Specimen preparation

Three 1.9 ml samples of each platelet apheresis donation were collected in Eppendorf tubes under sterile conditions, following gentle blending. Out of these three samples, one was used for the determination of NO levels. A total of 100 μl DMSO (Sigma-Aldrich, St. Louis, MO, USA) was added to the remaining two samples, respectively, which were then placed on a level oscillator, with an oscillation frequency of 60–70 times/min. The final concentration of the DMSO was 5%. Following this, 3.4 μl GSNO (1 mg/ml, fresh) was added to one of the two samples, at a final concentration of 10 μM. The two samples were then balanced at 22°C for 10 min and rapidly cryopreserved at −80°C. One week later, the two samples, i.e. the frozen platelet group (DMSO-treated) and the GSNO group (DMSO+GSNO-treated) were removed and rapidly defrosted at 37°C in order to determine the aggregation rate, membrane glycoprotein expression and NO content.

### Quantitative measurement of platelet aggregation

The platelet level in each group was adjusted to (2–3)x10^6^/ml with the original plasma. The reaction volume was set at 250 μl. Platelet-poor plasma (PPP) from the same sample was used as the substrate, followed by pressing the key of PPP. It was not necessary to pre-warm the PPP, and a magnetic stirring rod was not added to the PPP test cup. Instead, a magnetic rod was placed into the platelet-rich plasma (PRP) test cup, which was then added to the test channel and pre-warmed in the test area for 1 min. Following the prewarming, 10 μl (1 mmol/l) adenosine diphosphate (ADP; Sigma-Aldrich) was added to each test cup with a micropipette. An SC-2000 platelet aggregation instrument (Succeeder Technology Development Co., Ltd., Beijing, China) was used to measure the platelet aggregation.

### Determination and calculation of the NO content of platelets

An NO kit (comprising nitrate reductase) was purchased from Nanjing Jiancheng Technology Co., Ltd. (Nanjing, China). The detector tubes were divided into three groups: blank, standard and test tubes. The components in each tube are displayed in Table I. The components were added to each tube and mixed at 37°C in a water bath for 60 min. Reagents were added to each tube and fully vortically blended for 30 sec. The tubes were then left to stand at room temperature for 40 min, prior to being centrifuged at 2,740–3,580 × g for 10 min. Following this, 0.5 ml supernatant was blended into 0.6 ml chromogenic agent and left to stand at room temperature for 10 min. Distilled water was used for zero adjustment. The absorbance value in each tube was measured at 550 nm, with a light path of 0.5 cm.

The content of NO (μmol/l) was calculated according to the following formula, based on the measured absorbance values of the various tubes: NO content=[(absorbance of test tube-absorbance of blank tube)/(absorbance of standard tube-absorbance of blank tube)] × concentration of standard substance.

### Determination of platelet membrane glycoprotein expression

Peridinin chlorophyll protein complex (PerCP)-labeled anti-CD61, fluorescein isothiocyanate (FITC)-labeled PAC-1, phycoerythrin (PE)-labeled CD62P, allophycocyanin (APC)-labeled CD42b,, PE-labeled immunoglobulin G (IgG; negative control for CD62P) and APC-labeled IgG (negative control for CD42b) antibodies were purchased from Becton Dickinson (BD Biosciences, Franklin Lakes, NJ, USA). RGDS, as a blocker for PAC-1, combining with PAC-1, was used as negative control for PAC-1.

Labeled antibody were mixed for control tube and test tube. Mixed antibody (20 μl) and 10 μl platelets were added to each test tube, in accordance with Table II, while 20 μl mixed antibody and 10 μl platelets were added to each control tube. The tubes were kept away from the light at room temperature for 15 min, prior to the addition of 500 μl 1% paraformaldehyde to the tubes. The tubes were then stored away from the light at 4°C.

The analysis of the results from the four-color flow cytometry was performed as follows: Data were obtained from the cytometer and CellQuest software (BD Biosciences) was opened following startup. The control and test tubes were placed in the correct order and the condition of access (CD61-PerCP positivity) was chosen. The log pattern was selected in the forward scatter detector (FSC) and the side scatter detector (SSC), and the photomultiplier tubes (PMTs) of first fluorescence (FL1) and second fluorescence (FL2) were adjusted using the control tubes. The negative population was located on the lower left corner of the FL1 versus FL2 dot plot. The FL2-FL1 and FL1-FL2 compensations were adjusted using PAC-1 FITC/IgG PE/CD61 PerCP and PAC-1 FITC+RGDS/CD62P PE/CD61 PerCP, respectively. The cytometry data of various tubes, the contents of which are presented in Table II, were obtained.

The platelet group was identified for gating in the CD61 versus SSC dot plot. The CD61 versus SSC dot plot displayed three groups, i.e., the CD61-positive/low-SSC group (predominantly composed of platelets), the CD61-positive/high-SSC group (predominantly composed of blood cells adhered to platelets) and the CD61-positive/lower-scattered light group (predominantly composed of platelet-derived fragments). Since the size and the graininess of the platelet and red blood cell groups are similar under physiological and pathological conditions, it was not recommend that the FSC-SSC plot was used for gating. Therefore, the platelet group was identified for gating in the CD61 versus SSC dot plot (platelets and platelets adhered to white blood cells). The two-parameter analysis of PAC-1 FITC versus CD62P PE was performed inside the gating to obtain statistical results.

### Statistical analysis

The data are presented as the mean ± standard deviation. All statistical data were analyzed using SPSS 17.0 software (SPSS, Inc., Chicago, IL, USA). The results of NO content, aggregation rate and expression of membrane glycoproteins for the three groups of platelets (fresh liquid blood platelets, frozen platelets and frozen platelets treated with GSNO) were compared. Comparisons were evaluated using an independent sample t-test. P<0.05 was considered to indicate a statistically significant result.

## Results

### Platelet aggregation rate

As demonstrated in Table III, the ADP-induced platelet aggregation rate was 63.44±2.96 and 35.47±2.93% in the fresh liquid platelet and frozen platelet groups, respectively. The result for the frozen platelet group was significantly lower than that for the fresh liquid platelet group (P=0.000). The ADP-induced platelet aggregation rate was 24.43±3.07% in the GSNO-treated frozen platelet group, which was significantly lower than that in the other two groups. The results indicate that DMSO and GSNO exerted certain protective functions in platelet aggregation, which was reflected in the weakening of the aggregation response to the ADP-induced polymerization.

### NO level in platelets

Statistical analysis revealed that the NO level in the fresh liquid platelets from 32 normal blood donors was 31.59±16.88 μmol/l, whereas the NO level in the frozen platelets from 32 normal blood donors was 22.16±6.38 μmol/l. The NO level in the GSNO-treated frozen platelets from 32 normal blood donors was 45.64 6.31 μmol/l (Table IV).

As demonstrated in Table IV, the NO level in the fresh liquid platelet group was significantly higher compared with that in the frozen platelet group (t=2.958, P=0.004), whereas the NO level in the fresh liquid platelet group was significantly lower compared with that in the GSNO-treated frozen platelet group (t=4.289, P=0.000).

### Platelet flow cytometry

**Figure 1:**
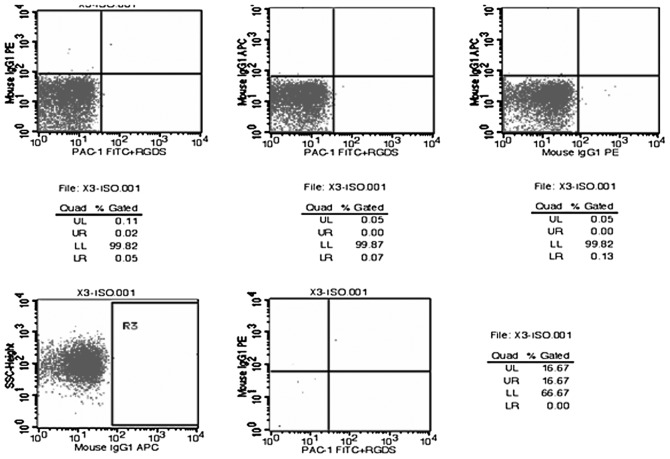
Flow cytometry charts of the negative controls for PAC-1, CD62P and CD42b, respectively. RGDS, Arg-Gly-Asp-Ser; FITC, fluorescein isothiocyanate; IgG, immunoglobulin G; PE, phycoerythrin; APC, allophycocyanin.

**Figure 2:**
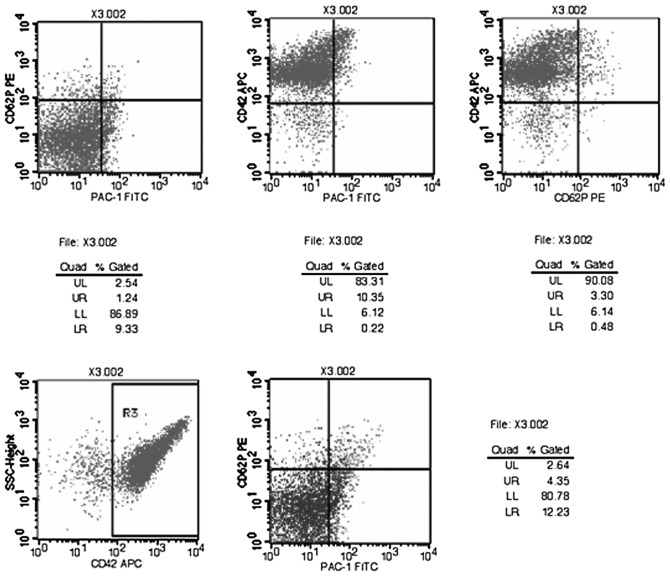
Flow cytometry charts for random samples of fresh liquid platelets. The cross marker on the first graph was decided by the combination of CD62P-phycoerythrin (PE) and PAC-1-fluorescein isothiocyanate (FITC) monoclonal antibodies. The active markers of platelets are CD62P positive and PAC-1 negative in the UL quadrant, CD62P and PAC-1 positive in the UR quadrant, CD62P and PAC-1 negative in the LL quadrant and CD62P negative and PAC-1 positive in the LR quadrant, respectively. APC, allophycocyanin.

**Figure 3:**
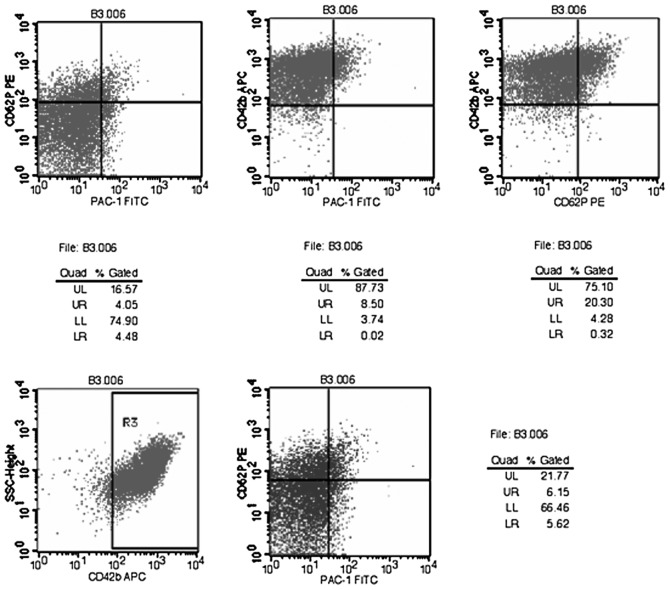
Flow cytometry charts for random samples of frozen platelets. FITC, fluorescein isothiocyanate; PE, phycoerythrin; APC, allophycocyanin.

**Figure 4:**
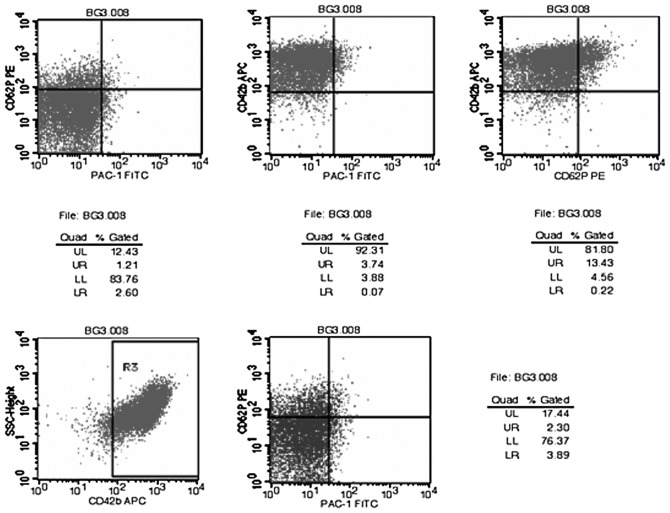
Flow cytometry charts for random samples of frozen platelets treated with S-nitrosoglutathione (GSNO). PE, phycoerythrin; APC, allophycocyanin; FITC, fluorescein isothiocyanate.

Flow cytometry charts for the tests of random samples of platelet membrane glycoprotein molecules are displayed in Figs. 1–4. The results of the flow cytometric analysis of the fresh liquid platelet, frozen platelet and GSNO-treated frozen platelet groups are displayed in Table V.

The differences in membrane glycoprotein expression in the fresh liquid platelet, frozen platelet and GSNO-treated frozen platelet groups (groups 1, 2 and 3, respectively) are demonstrated in Table V. There were no significant differences in the single-positive PAC-1 expression among the three groups (comparison between groups 1 and 2, P=0.743; comparison between groups 1 and 3, P=0.473; comparison between groups 2 and 3, P=0.297). However, the single-positive CD62P expression in group 1 was significantly lower than that of groups 2 and 3 (P=0.000 and P=0.001, respectively), although there was no significant difference between groups 2 and 3 (P=0.165). Furthermore, the expression of single-positive CD42b in group 1 was significantly higher than that of groups 2 and 3 (P=0.007 and P=0.015, respectively), but no significant difference was observed between groups 2 and 3 (P=0.778).

There were no significant differences in the dual-positive PAC-1+CD62P expression among the three groups (group 1 versus group 2, P=0.125; group 1 versus group 3, P=0.765; group 2 versus group 3, P=0.165), or in the dual-positive PAC-1+CD42b expression (group 1 and versus group 2, P=0.793; group 1 versus group 3, P=0.424; group 2 versus group 3, P=0.290). However, the dual-positive CD62P+CD42b expression in group 1 was significantly lower to that in groups 2 and 3 (P=0.000 and P=0.000, respectively). There was no significant difference between groups 2 and 3 (P=0.207).

There were no significant differences in tri-positive PAC-1+CD62P+CD42b expression among the three groups (group 1 versus group 2, P=0.114; group 1 versus group 3, P=0.815; group 2 versus group 3, P=0.176).

## Discussion

The enhancement of *in vivo* hemostatic function in cryopreserved platelets may be correlated with changes in the expression of platelet membrane glycoproteins, while the inhibition of platelet aggregation in cryopreserved platelets by GSNO may be associated with alterations in the NO levels. The current study was conducted to further investigate changes in platelet membrane glycoprotein expression.

The results of the present study revealed that with regard to the expression of platelet membrane glycoproteins in the fresh liquid platelet, frozen platelet and GSNO-treated frozen platelet groups, the differences in the expression of PAC-1 were not statistically significant, i.e. there were no significant differences in the single-positive PAC-1, dual-positive PAC-1+CD62P, dual-positive PAC-1+CD42b and tri-positive PAC-1+CD62P+CD42b expression levels among the three groups. However, the CD62P expression was significantly lower in the fresh liquid platelet group compared with that in the frozen platelet and GSNO-treated frozen platelet groups (P=0.000 and P=0.001, respectively), and the CD42b expression in the fresh liquid platelet group was significantly higher than that in the frozen platelet and GSNO-treated frozen platelet groups (P=0.007 and P=0.015, respectively). Furthermore, the CD62P+CD42b expression in the fresh liquid platelet group was significantly lower than that in the frozen platelet and GSNO-treated frozen platelet groups (P=0.000 for each). However, the expression of platelet membrane glycoproteins in the frozen platelet group was not significantly different from that in the GSNO-treated frozen platelet group.

When resting platelets are activated, the platelet membrane glycoproteins rapidly undergo changes in number and structure. The changes in the platelet membrane are of primary importance in altering platelet survival. At the time of storage, the platelet may be activated, injured and cleared (6). Avoiding platelet activation may improve the recovery rate and prolong the survival time. The injury occurring to platelets as a result of preservation has been considered an important factor leading to the failure of platelets following long-term preservation. Platelet injury during preservation has been revealed to be correlated with *in vitro* platelet activation, i.e., the higher the degree of *in vitro* platelet activation, the more serious the platelet preservation injury (6). The presence of the glycoprotein GPIIb/IIIa complex is currently accepted as the ideal evaluation index of platelet activation. Platelet aggregation function is an important factor that is closely associated with the platelet hemostatic effect, and is primarily dependent on the quality and quantity of membrane glycoproteins GPIIb/IIIa present on the surface of the platelets. Platelet aggregation rate is an important indicator reflecting the efficacy of the platelet aggregation function.

PAC-1, a type of monoclonal antibody that binds to activated human platelets, is only able to combine with the GPIIb/IIIa compound present on the activated platelet. In the present study, flow cytometry was used to detect the positive expression level of PAC-1, which represented the degree of GPIIb/IIIa activation and reflected the early activation of the platelets.

Platelet apheresis itself is an important factor affecting the clinical effect of platelet transfusions. Our results demonstrated that an extended storage time did not significantly alter the level of PAC-1 expression, a platelet activation marker, in the frozen platelet or GSNO-treated frozen platelet groups (P>0.05).

During *in vitro* platelet activation, GPIIb/IIIa translocating from within the platelet to the platelet surface is degraded by proteases of the immune system, resulting in the loss of normal aggregation function. By contrast, the GPIIb/IIIa stored inside the platelets is able to continue to transfer to the platelet surface to enhance the GPIIb/IIIa levels, thus maintaining the GPIIb/IIIa presence and the immediate hemostatic response. However, the finite nature of the GPIIb/IIIa storage leads to a decline in the polymerization capacity of the activated platelets in response to the aggregation inducer. Therefore, while the immediate hemostatic function is maintained, eventually, the hemostatic function declines following platelet transfusion (18).

There are numerous membrane glycoproteins in normal human platelets, of which GPIb/IX and GPIIb/IIIa are the major glycoproteins. Each platelet comprises ~25,000 GPIb/IX molecules. GPIb consists of an αβ chain linked by disulfide bonds, with an Mr of 165,000. The platelet membrane glycoprotein, GPIbA, is one of the components of the GPIb/IX complex, and participates in numerous physiological and pathological processes. It is the receptor for the adhesive protein von Willebrand Factor and for thrombin, in addition to being one of the key substances mediating the initial contact between platelets and the vascular walls, and participating in platelet adhesion, early physiological hemostasis and pathological thrombosis (7).

During *in vitro* platelet activation in the frozen platelet and GSNO-treated frozen platelet groups, the proteases of the immune system may have damaged the GPIb on the surface of the platelets, resulting in a loss of normal function (2). It was observed in the current study that the expression of CD42b in the fresh liquid platelet group was significantly different from that in the frozen platelet and GSNO-treated frozen platelet groups, and that the expression of CD62P+CD42b in the fresh liquid platelet group was significantly different from that in the frozen platelet and GSNO-treated frozen platelet groups. In addition, the polymerization capacity of the platelets, induced in response to an aggregation inducer, was reduced, eventually leading to decline in hemostatic function following platelet transfusion.

*In vitro*, platelets may be naturally activated at room temperature, leading to the irreversible expression of high levels of CD62P on the platelet membrane; therefore, CD62P is considered to be the best marker and gold standard of platelet activation. Furthermore, CD62P exists solely on the surface of activated platelets, a quality making CD62P an ideal indicator in the detection of platelet activation. As such, CD62P is attracting an increasing focus. A high expression level of CD62P in activated platelets may be sensitively and specifically detected using flow cytometry (8). It was revealed in the current study that the expression of CD62P in the fresh liquid platelet group was significantly different to the expression in the frozen platelet and GSNO-treated frozen platelet groups and that the expression of CD62P+CD42b in the fresh liquid platelet group was significantly different from the expression in the frozen platelet and GSNO-treated frozen platelet groups. Furthermore, the polymerization capacity of the platelets, induced in response to an aggregation inducer, was decreased when CD62P was activated, eventually leading to a decline in hemostatic function following platelet transfusion. However, the immediate hemostatic function was maintained due to the fact that the activation of GPIIb–IIIa was inhibited in the frozen platelets and GSNO-treated frozen platelets.

The results of this study revealed that the NO level in the fresh liquid platelet group was significantly higher compared with that of the frozen platelet group (t=2.958, P=0.004), whereas the NO level in fresh liquid platelet group was significantly lower compared with that of the GSNO-treated frozen platelet group (t=4.289, P=0.000). The level of NO in the fresh liquid platelet group, which was markedly higher than that in the frozen platelet group, may have prevented platelet consumption and maintained platelet function. However, the level of NO in the fresh liquid platelet group was significantly lower than that in the GSNO-treated frozen platelet group. Due to the presence of the external NO donor in the GSNO-treated frozen platelet group, NO may have been released to improve the inhibition of platelet activation and aggregation, and to maintain the platelet function. Although there was a decline in the metabolic function of the frozen platelets, NO loss was still apparent in the cryopreservation process. The NO content may therefore be increased by adding an external NO donor.

In a cardiopulmonary bypass model (9), an external NO polymer, acting as an NO donor, was able to release NO to prevent platelet consumption and maintain platelet function.

Although NO is important in many biological processes, <10% of the studies in the last century in the field of NO mention the direct measurement of NO. By contrast, the NO content is often indirectly measured, for example, by the measurement of S-nitrosothiols in body fluids using chemiluminescence methods (10). A type of porphyrin microsensor has also been used for the measurement of NO released by whole blood platelets and washed platelet suspensions. The use of these techniques has demonstrated that NO is produced during the aggregation of human platelets (11).

The current technologies that are used for the measurement of NO (12) include the chemiluminescence and Griess methods, paramagnetic resonance spectroscopy, paramagnetic resonance imaging, spectrophotometry and biological assays. Each technology has certain advantages; however, there are also several shortcomings, such as low sensitivity and a requirement for complex and expensive laboratory equipment.

S-nitrosothiols are formed by thiols through S-nitrosyl acylation, in the presence of NO or NO_2_, and have been demonstrated to be effective inhibitors of *in vitro* platelet aggregation. It has been shown that the stability of highly reactive and unstable NO may be maintained through reactions with carrier molecules, such as R-SH, thereby prolonging the half-life of NO and maintaining its biological activity. As a stable S-nitrosothiol, GSNO may be synthesized by the most abundant intracellular thiol, glutathione, in order to maintain a dynamic equilibrium. As an NO donor, GSNO has the potential to be added to cryopreserved platelets in order to release NO and inhibit the adherence of platelets to collagen, thereby inhibiting platelet activation and maintaining platelet function (3).

The current methods for the detection of platelet function include platelet aggregation instruments for the detection of platelet aggregation and bleeding time and enzyme-linked immuno-sorbent assay (ELISA) for the detection of thromboxane B2 (TXB2). In the present study, flow cytometry was directly used for the detection of platelet membrane glycoproteins on the surface of platelets. This was a good method of detecting platelet activation as it analyzed the platelets in close to their natural preservation state, in addition to being easy to operate. Furthermore, the procedure minimized the changes in platelet status.

The results revealed that the ADP-induced platelet aggregation rate in the frozen platelet group was significantly lower than that of the fresh liquid platelet group, and that the ADP-induced platelet aggregation rate in the GSNO-treated frozen platelet group was significantly lower than that in the fresh liquid platelet and frozen platelet groups. These observations suggest that DMSO and GSNO played certain protective roles in platelet aggregation, which was reflected in the reduction of the aggregation response, due to inhibition of the polymerization induced by ADP.

An NO-releasing polymer has been demonstrated to inhibit platelet activation (13). It was observed that NO released by the polymer was able to reduce platelet activation and reduce thrombosis by affecting the membrane glycoprotein, P-selectin. NO may lead to molecular changes in the platelet membrane glycoproteins and inhibit thrombosis, indicating that NO-releasing polymers may be used to coat ECCs for biological and medical equipment.

NO may inhibit platelet function through the precise regulation of the platelet response and the inhibition of platelet activation via the NO-soluble guanylyl cyclase (sGC)-cGMP signal pathway (14). NO released by the endothelium may activate the only receptor of NO that is located inside platelets (NO-sensitive sGC), produce cGMP and activate cGMP-dependent protein kinase (PKG), leading to a reduction in intracellular calcium levels and inhibiting platelet adhesion and aggregation (14). In one study, the inhibitory effect of NO donor substances on platelet activation was observed to be sGC-dependent only at the micromolar, but not at the millimolar, concentration level (15).

Non-cGMP dependent mechanisms, such as S-nitrosylation, have also been suggested as alternative NO-mediated signaling pathways. GSNO, an NO donor, has been demonstrated to inhibit the adherence of platelets to static collagen in a concentration-dependent manner. Biotin transformation analysis of platelets revealed that there were several S-nitrosylated proteins in the basic state. At concentrations sufficient to inhibit platelet adhesion, the treatment of platelets with an exogenous NO donor was able to increase the types of S nitrosylation and led to the hyper-S-nitrosylation of S-nitrosylated proteins. The degree of S-nitrosylation caused by exogenous NO was not affected by platelet activation. Furthermore, in the absence of exogenous NO, platelet activation did not increase S-nitrosylation, and the nitrocellulose level remained below the basic level, indicating that platelet-derived NO was not able to induce this type of protein modification. The S-nitrosylation of platelet proteins induced by exogenous NO may be an important non-cGMP-dependent signaling mechanism, which may regulate platelet adhesion (3).

S-Nitrosothiols, such as GSNO, have numerous potential clinical applications, particularly as anti-thrombotic agents, primarily due to their platelet inhibitory effect and the fact that they exhibit a certain degree of platelet selectivity. A recent study revealed that S-nitrosothiols are involved in a variety of pathways. The delivery of an NO-related signal into cells by a stable S-nitrosothiol compound was demonstrated to result in the denitrification of cell surface enzymes, in addition, to the transportation of intact S-nitroso-cysteine by the amino acid transport system (L-AT). The different roles of these pathways in platelets and vascular cells may partially explain the selective effects on platelets (16).

Low levels of GSNO have been demonstrated to inhibit platelet aggregation without leading to vasodilation, indicating that the action of NO is platelet-selective; this selectivity may involve mercaptan isomerase on the surface of cells, and particularly the protein disulfide isomerase (csPDI; EC 5.3.4.1). In a previous study, flow cytometry demonstrated that the positive expression rate of csPDI in platelets was higher than in blood vessel cells. Furthermore, the reductase activity associated with mercaptan isomerase was higher on platelets (P<0.01). Following the activation of the cell, the activities of csPDI on the platelets and smooth muscle cells were increased; however, the activity on the endothelial cells was not increased. GSNO released NO more inside the platelet cells than inside the vascular cells (P<0.002). Compared with the vessel wall cells, the activity of mercaptan isomerase on the surface of the platelets was increased, which may explain the selective action of GSNO on platelets and aid the elucidation of its anti-thrombotic ability (17).

DMSO has been widely used in the field of cell biology, not only a cryoprotectant, but also as a fortifier of cell fusion and permeability. The protective effect of DMSO on platelets has been demonstrated to be better than that of other cryoprotectants, and the function of DMSO in platelet cryopreservation is not readily replicated by other cryoprotectants (18).

New preservation solutions have been developed, which may, alone or in combination with other preservation solutions, improve frozen platelet function. These solutions have been expected to further improve the clinical infusion effects of cryopreserved platelets. However, the inhibition of the activation of cryopreserved platelets is challenging. The results of the present study indicated that the expression of platelet membrane glycoproteins in the GSNO-treated frozen platelet group was not significantly different from that of the frozen platelet group. Treatment with GSNO may have altered the molecular arrangement and structure of the frozen platelets, or affected the platelets through other mechanisms.

## Figures and Tables

**Table I:** Components added to each of the tubes.

	Type of tube		
	
Component	Blank	Standard	Test
Double-distilled water (ml)	0.1	-	-
100 μmol/l standard solution (ml)	-	0.1	-
Sample (3 types of platelets, ml)	-	-	0.1
Mixing reagent (ml)	0.4	0.4	0.4

**Table II:** Combinations of fluorescent antibodies in the control and test tubes.

	Quantity added (μl)						
	
Tube	CD61 PerCP	PAC-1 FITC	CD62P PE	CD42b APC	RGDS	IgG PE	IgG APC
Control tube	100	50	-	-	50	100	100
Test tube	100	100	100	100	-	-	-

RGDS, Arg-Gly-Asp-Ser; IgG, immunoglobulin G; PerCP, peridinin chlorophyll-protein complex; FITC, fluorescein isothiocyanate; PE, phycoerythrin; APC, allophycocyanin.

**Table III:** Aggregation of the three groups of platelets.

Group	n	Aggregation (%)
Fresh liquid platelets	12	63.44±2.96
Frozen platelets	12	35.47±2.93^a^
Frozen platelets treated with GSNO	12	24.43±3.07^b^

Results are presented as the mean ± standard deviation.

a P=0.000,

b P=0.000 compared with fresh liquid platelets.

GSNO, S-nitrosoglutathione.

**Table IV:** NO concentration of the three groups of platelets.

Group	n	NO concentration (μmol/l)
Fresh liquid platelets	32	31.59±16.88
Frozen platelets	32	22.16±6.38^a^
Frozen platelets treated with GSNO	32	45.64±6.31^b^

Results are presented as the mean ± standard deviation.

a P=0.004,

b P=0.000 compared with fresh liquid platelets.

NO, nitric oxide; S-nitrosoglutathione (GSNO).

**Table V:** Flow cytometry data of platelets in the three platelet groups.

Group	PAC-1 single-positive	CD62P single-positive	CD42b single-positive	PAC-1+CD62P dual-positive	PAC-1+CD42b dual-positive	CD62P+CD42b dual-positive	PAC-1+CD62P+CD42b tri-positive
Fresh liquid platelets	8.74±6.51	12.74±9.64	90.46±6.65	3.11±3.66	8.98±6.48	10.34±7.49	4.25±3.07
Frozen platelets	9.72±6.01	31.72±8.20	76.94±15.66	6.39±4.48	9.76±6.07	28.49±7.74	8.74±5.25
Frozen platelets with GSNO	6.60±3.48	26.34±9.97	78.32±12.54	3.75±2.39	6.58±3.49	23.99±9.26	4.91±2.32

Data are presented as the mean ± standard deviation.
